# Determinants of school attendance in elementary school students in Japan: a structural equation model

**DOI:** 10.1186/s13034-021-00391-5

**Published:** 2021-07-27

**Authors:** Hiromi Nakamura-Thomas, Nobuyuki Sano, Donald Maciver

**Affiliations:** 1 Graduate School of Health, Medicine and Welfare, School of Health, Medicine and Welfare, Saitama Prefectural University, 820 San-no-miya, Koshigaya, Saitama Japan; 2 Department of Occupational Therapy, Faculty of Medical Sciences, Fukuoka International University of Health and Welfare, 3-6-40 Momochihara, Sawara ku, Fukuoka city, Fukuoka Japan; 3 School of Health Sciences, Queen Margaret University, Queen Margaret University Way, Musselburgh, EH21 6UU UK

**Keywords:** School attendance, Friends, School teachers, Positive relationships, Elementary school, Japanese

## Abstract

**Background:**

Managing school nonattendance is a priority worldwide. Frequent school nonattendance in early school years has immediate and long-term negative effects. Although strategies to address nonattendance are being developed and implemented, the number of students with school nonattendance issues is increasing. In this study, we explored students’ feelings and perceptions about attending school and the potential determinants of a positive attitude towards attending school.

**Methods:**

We hypothesized that a positive perception towards attending school was influenced by relationships, perceptions of current circumstances, subjective health, and having someone to share experiences and thoughts with. For examining the hypothesized model, an original questionnaire with 14 items was developed, including perceptions towards school attendance (an item), relationships with friends and school teachers (5 items), current circumstances (4 items), subjective health (3 items), and the individuals available to share experiences and thoughts with (1 item). In total, 6860 children submitted the questionnaire (85.3% response rate) and 6841 responses were included to examine the model. Children were 10 or 11 years old, and selected from 111 state-run schools in 8 randomly selected school districts.

**Results:**

The final model demonstrated good fit and showed that the latent variable of relationships with friends and school teachers directly impacted on how children felt about attending school. The latent variable of subjective health also directly impacted on how children felt about attending school but not strongly. Other latent variables were not significant.

**Conclusions:**

The importance of positive relationships with friends and teachers in overcoming school nonattendance has been emphasized in previous studies. This study has provided evidence that these relationships impacted children’s positive perception about attending school in a large sample of students aged 10–11 years. The latent variable of subjective health may require more items to capture mental health.

**Supplementary Information:**

The online version contains supplementary material available at 10.1186/s13034-021-00391-5.

## Introduction

Frequent school nonattendance in early school years has immediate and long-term negative effects on academic performance, graduation rates, social functioning, job opportunities, and health [1–4]. As school nonattendance has long-term negative effects, it is a public health and educational issue [1]. Therefore, managing school nonattendance is a core theme in educational policy around the world [5]. A systematic review reported that chronic school nonattendance appeared to be driven by overlapping medical, individual, family, and social factors, including poor health, bullying, perceived lack of safety, and family influences [1]. For some children, school can be a source of frustration, leading to avoidance behaviors, problematic relationships, and stress [2–4].

Poor mental health is known as a risk for school nonattendance. Systematic reviews have identified the importance of identifying anxiety or depression among children and adolescents [6, 7]. School climate is considered to be a significant determinant of children’s mental health [8, 9] and behavior [10]. Within the environment, relationships with peers and school teachers have been found to be important [8–10]. Such relationships with peers and teachers are also key elements for supporting students’ school attendance. Having a positive peer relationship with classmates is important during the elementary school years. Elementary school children who are not liked by their classmates or who have no or only a few friends are at risk of bullying [11, 12] with associated emotional and behavioral needs [13–16]. Explicit facilitation of peer-to-peer understanding, acceptance, play and friendships are recommended [17]. Teachers can support mental health among elementary school children [18]. Thus, children’s access to supportive adults, particularly teachers, is important [19–24].

Different strategies have been implemented for solving or preventing school nonattendance, with varying degrees of success. A project following children from preschool to elementary school for 8 years, including a collaboration between kindergarten teachers, local government offices, school psychologists and public health nurses succeeded in reducing the number of students refusing to attend school in Japan [25]. Free school meals can support school attendance among students from low-income families [26]. After-school programs have been provided for students who are at risk of school nonattendance; however, a meta-analysis found unclear evidence of effectiveness [27].

Whilst strategies to manage school nonattendance may be applied by most school systems, including emotional and practical support, as well as school-wide and teacher training, the rate of students with school nonattendance is increasing [17]. School nonattendance is defined by the Japanese state as nonattendance at school for a period of more than 30 days per year without a health or financial cause [17]. A recent nationwide survey identified more than 31,000 elementary school children who had not attended school for over 30 days (0.5% of the total school population) [17]. School teachers are responsible for a wide range of students in the classroom and their workload is increasing. They may not have the time or knowledge to provide individualized support. Specialist providers may also be required. School counselors are one such resource and have been newly introduced in Japan to support children [17]. In most cases, school counselors support children with more complex social, emotional or behavioral issues [28].

Japan also has some unique characteristics that may contribute to outcomes, including homogeneity, gender differences and social characteristics that may influence attendance. People in a homogeneous society, such as Japan are more likely to think and behave as other people in the group do. An elementary school child may feel different and/or be bullied or discriminated against because of perceived differences [29]. Research shows an important age effect. Nonattendance increases in the 4th or 5th year of primary elementary education [17]. It has been proposed that the introduction of abstract concepts and theory may be a factor [30]; however, the exact casual mechanism remains unknown.

There are state-run and private schools in Japan. State schools adhere to government guidelines, while private schools may implement different policies based on the school’s individual philosophy. In societies that value conformity and homogeneity, such as Japan, students are strongly encouraged to go to school, irrespective of their feelings or wishes. A recent nationwide survey revealed that 3.3% of 6450 elementary school students did not want to go to school [31]. The educational board in Saitama, Japan, intended to explore whether the current implementation of strategies to reduce nonattendance was effective. The educational board wanted to explore students’ feelings and perceptions about attending school and the potential determinants of a positive attitude towards attending school. The educational board had identified support for students with school nonattendance as a key priority and wanted to develop data and strategies to support school attendance. At the time of the research, there was no locally available, standardized instrument to measure how children felt about attending school. The school refusal assessment scale was proposed [32]; however, a Japanese version had not been developed. Therefore, we developed a new model and questionnaire that described the potential determinants of positive perceptions towards attending school among elementary school students in state-run schools and explored the model in this research.

## Methods

### Study design

The current study was a cross-sectional study of elementary school children in the 5th year in state-run schools in Saitama, Japan. We explored students’ feelings and perceptions about attending school and the potential determinants of a positive attitude towards attending school. We hypothesized that a positive perception towards attending school was influenced by relationships, perceptions of current circumstances, subjective health, and having someone to share experiences and thoughts with.

### Setting

This study was conducted collaboratively between Saitama Prefectural University and the Saitama educational board. The educational board manages 802 state-run elementary schools (99% of total elementary schools) in Saitama. Those state-run elementary schools are grouped into 72 school districts matched to municipalities. Saitama, located north of Tokyo, has the third largest area, the fifth highest population, and the fourth highest population density. Saitama government issued reports compare southern, northern, western, and eastern areas based on the population and tax revenue. Using the four area groups, we selected randomly 2 school districts from each area, thus, we targeted a total of 8 school districts. Chairpersons of the school districts provided permission to implement the survey. There were 2 chairpersons that did not wish to participate, thus, a second draw was completed to select the next school district. The 8 school districts included presented one ninth (11.1%) of 72 school districts, thus, we expected to obtain data from 89 state-run elementary schools (11.1%) out of 802 schools.

The original questionnaire described below was distributed between April and December 2019. Each school district chose the periods for administration. Prior to distribution, school district officers visited the schools, described the aim of this survey to school teachers, and obtained agreement to conduct the survey. Data collection was completed by teachers in each classroom.

### Participants

Children begin elementary education at 6 years of age in Japan. Thus, elementary school children in the 5th year were either 10 or 11 years old during the data collection period. There were 8045 eligible children who were in the 5th year for this survey from the selected 8 school districts (Additional file 1: Table S1). Unlike European countries with inclusive policies, children who require special support in school because of physical, psychological, and intellectual issues attend segregated special schools [17]. No school providing special education was included in this survey. For examining the hypothesized model with a structural equation model (SEM), described below, we expected to obtain data from approximately 6838 children (85% of the 8045 eligible children). Generally, SEM requires large sample sizes although no standardized guideline for sample size exists.

### Variables

**Fig. 1:**
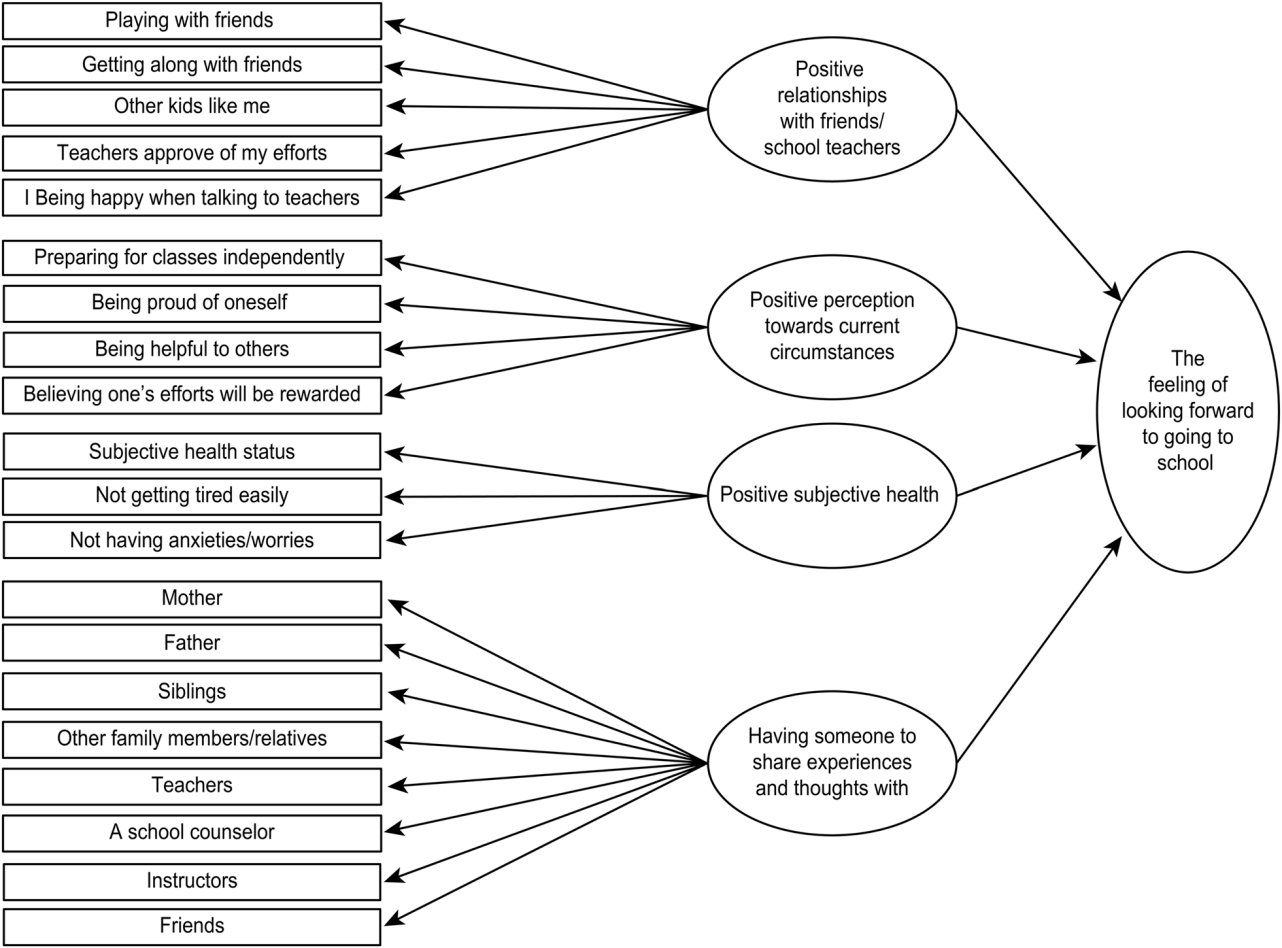
Hypnotized model

Variables and the potential relationships among variables were identified based on previous studies [1–16, 18–24] and Japanese national guidelines [17]. Key areas of interest included relationships with peers, relationships with school teachers, communication, sharing of experiences and emotions, and health conditions or health problems. Based on this review, we hypothesized that a positive perception towards attending school was influenced by positive relationships with friends and school teachers, positive perceptions of current circumstances, positive subjective health, and having someone to share experiences and thoughts with (Fig. 1).

To examine the model, an original questionnaire was developed through a collaboration between members of the Saitama educational board and faculty members of Saitama Prefectural University. Those members’ specialty was education. The questionnaire was piloted in 100 students by the faculty member who initiated the project to confirm the face-validity and interpretability of the questions. Several meetings, including face-to-face discussion, videoconference and email discussion, were held between the members of the development team to finalize the questionnaire. Wordings for questions were refined based on the literature, guidelines issued by the educational board, and the expertise of the development team. It was strongly felt by the development team, given their experience working in and around schools, that a large number of questions would be a burden to the participants; thus, a shorter tool was desirable.

### Measurement

There were 14 items in the original questionnaire. The first 13 items targeted intrapsychic and interpersonal determinants of positive perceptions towards attending school. The 14th item listed potential persons who were accessible for the participants to share their experiences.

To capture how children felt about attending school, the item wording was “I am looking forward to going to my school”. The responses were 4 = strongly agree, 3 = agree, 2 = disagree, and 1 = strongly disagree.

To capture positive relationships with friends and school teachers, the following five items were positively worded: “I play with friends a lot”, “I get along well with friends”, “Other kids like me”, “School teachers approve of my efforts” and “I am happy when talking to school teachers”. For the five items, the responses were 3 = very much, 2 = a little bit, and 1 = not at all.

To capture the positive perceptions of current circumstances, the following four items were positively worded: “I am able to prepare for classes by my own”, “I am proud of myself”, “I am helpful to others” and “I believe my efforts will be rewarded”. For the item about being able to prepare for classes independently, the responses were 4 = very well, 3 = fairly, 2 = poorly, and 1 = not at all. For other items, the responses were 3 = very much, 2 = a little bit, and 1 = not at all.

To capture subjective health, the following three items were employed: “My health status”, “I get tired easily” and “I have anxieties and/or worries”. For the general health status, the responses were 4 = good, 3 = fair, 2 = not very good, and 1 = not good at all. For the item for tiredness, the responses were 3 = not tired at all, 2 = sometimes tired, and 1 = frequently tired. For the item about anxieties and/or worries, the responses were 3 = no anxiety/worry at all, 2 = a little bit of anxiety/worry, and 1 = a lot of anxiety/worry.

To capture the persons with whom the children shared their experiences and thoughts as the 14th item, the following eight responses were selected: (a) my mother, (b) my father, (c) my siblings, (d) other family members/relatives, (e) school teachers, (f) a school counselor, (g) instructors of extracurricular activities, and (h) friends. The item also asked the participants the frequency of accessing those persons. The responses were 3 = frequently, 2 = sometimes, 1 = rarely, and 0 = there is no such person.

### Ethics

The research protocol was reviewed and approved by the Research Ethics Committee at Saitama Prefectural University (No. 19078).

### Statistical methods

The statistical data analysis included descriptive statistics, Cramer’s V, Spearman’s rank correlation coefficient, and a SEM. The analysis was conducted with HAD 17.0 [33], SPSS v.26 for Japanese (IBM, Japan), and *M*plus version 7.3 [34].

Before conducting the SEM, we observed the data characteristics. Because the data were collected from 8 school districts, we explored individual-level variance and group-level (school district-level, in this study) variance [35]. Group homogeneity was identified with intraclass correlation coefficient (ICC). A high ICC results in a biased error variance in conventional regression models, overestimating the relationship between variables. An ICC of 0.25 and higher indicates that much of the variation in the dependent variables is due to the features of groups rather than the characteristics of individuals [36, 37]. We employed a more stringent value of 0.001 for a significant difference.

Additionally, we observed sample descriptive data with individual-level variance. We compared boys and girls according to the survey items. For the comparisons, we used Cramer’s *V*, which indicates how strongly two categorical variables are associated, with 1 indicating a strong association and 0 indicating no association. Values of 0.1, 0.3, and 0.5 are considered to be small, medium, and large effect sizes, respectively [38]. We also used Spearman’s rank correlation coefficient between the variables. Coefficients were interpreted as limited (0.00 to 0.25), fair (0.25 to 0.50), moderate (0.50 to 0.75) and excellent (0.75 to 1.0) [39].

SEM with ordinal data was conducted using the modified weighted least squares method (WLSMV). We examined the hypothesized model using all data obtained by the original questionnaire and then, modified the model. The model fit indices were comparative fit index (CFI), Tucker Lewis index (TLI), and root mean square error of approximation (RMSEA). For CFI and TLI, a value higher than 0.9 is the best model fit. For RMSEA, a value of 0.05 and smaller is a close fit, a value of 0.08 and smaller is a reasonable fit, and a value of 0.1 and higher is a poor fit [40]. The RMSEA value was supplemented with a 90% confidence interval (90% CI).

## Results

### Participants

The average response rate was 85% (between 78.9% and 95.4% in school districts) (Additional file 1: Table S1). In total, 6860 children completed the questionnaire (the response rate was 85.3%). Among the collected data from 6860 children, 19 children were excluded because of responding to two items and fewer. As a result, data from 6841 children were analyzed. Among them, 2995 (43.78%) were boys, 3169 (46.32%) were girls, and 677 (9.9%) were children of uncategorized gender. Across the school district, no significant difference was observed in percentages between boys and girls (0.032 for Cramer’s *V*, *p* = 0.521). Every student was either 10 or 11 years old.

### Descriptive data

Table 1 shows the values for the valid sample number, interclass correlation, and *p*-value for each question item. Across the question items, the valid data rates were 95% and higher. No question item showed an ICC value of 0.05 or higher, indicating that all data could be described with individual-level variance.

**Table 1:** Values in valid sample, interclass correlation, and reliability for each question item (n = 6841)

Item wordings	Valid sample		ICC		
	n	%		95% CI Lower	95% CI Upper
1. I am looking forward to going to my school	6531	95.5	0.019*	0.008	0.076
2. I play with friends a lot	6786	99.2	0.009*	0.003	0.039
3. I get along well with friends	6777	99.1	0.003*	0.001	0.015
4. Other kids like me	6744	98.6	0.010*	0.003	0.041
5. School teachers approve of my efforts	6757	98.8	0.008*	0.003	0.036
6. I am happy when talking to school teachers	6766	98.9	0.011*	0.004	0.048
7. I can prepare for classes by my own	6525	95.4	0.009*	0.003	0.038
8. I am proud of myself	6771	99.0	0.008*	0.003	0.034
9. I am helpful to others	6759	98.8	0.008*	0.003	0.034
10. I believe my efforts will be rewarded	6767	98.9	0.003*	0.000	0.014
11. My health status is	6615	96.7	0.031*	0.012	0.115
12. I get tired easily	6708	98.1	0.006*	0.002	0.026
13. I have anxieties/worries	6757	98.8	0.021*	0.008	0.083
Persons I talk to when sharing my experiences and thoughts with					
a. My mother	6763	98.9	0.011*	0.004	0.046
b. My father	6696	97.9	0.007*	0.002	0.029
c. My siblings	6670	97.5	0.014*	0.005	0.056
d. Other family members/relatives	6732	98.4	0.019*	0.007	0.075
e. School teachers	6933	98.4	0.029*	0.012	0.109
f .A school counselor	6655	97.3	0.015*	0.006	0.062
g. Instructors of extracurricular activities	6741	98.5	0.004*	0.001	0.020
h. My friends	6100	98.9	0.014*	0.005	0.057

*ICC* interclass correlation, *CI* confidence interval

^*^ *p* < 0.001

### Reponses characteristics

Table 2 shows the response characteristics according to response alternatives in each item. For the item “I am looking forward to going to my school”, 45% of the children responded “agree”, followed by “strongly agree” (35%), “disagree” (14%) and “strongly disagree” (5%).

**Table 2:** Response characteristics according to response alternatives in items 1–13

Items	Valid response N	Response alternatives N (%)			
		Strongly agree	Agree	Disagree	Strongly disagree
1. Looking forward to going to my school	5918	2091 (35.33)	2686 (45.39)	852 (14.10)	289 (4.88)
		Very much	A little bit	Not at all	
2. Playing with friends a lot	6118	4269 (69.78)	1518 (24.81)	331 (5.41)	
3. Getting along well with friends	6112	5106 (83.54)	934 (15.28)	72 (1.18)	
4. Other kids like me	6082	2218 (36.47)	2985 (49.08)	879 (14.45)	
5. School teachers approve of my efforts	6097	2216 (36.36)	3111 (51.05)	767 (12.59)	
6. Being happy when talking to school teachers	6101	2716 (44.52)	2515 (41.22)	870 (14.26)	
		Vary well	Fairly	Poorly	Not at all
7. Preparing for classes by own	5912	1798 (30.40)	3398 (57.48)	631 (10.67)	86 (1.45)
		Very much	A little bit	Not at all	
8. Being proud of oneself	6102	2208 (36.18)	3001 (49.18)	893 (14.63)	
9. Being helpful to others	6090	2026 (33.27)	3241 (53.22)	823 (13.51)	
10. Believing one’s efforts will be rewarded	6098	4156 (68.15)	1726 (28.30)	216 (3.54)	
		Good	Fair	Not very good	Not good at all
11. Subjective health status	5983	4111 (68.71)	1621 (27.09)	224 (3.74)	27 (0.45)
		Not tired at all	Sometimes tired	Frequently tired	
12. Not getting tired easily	6057	1309 (21.61)	2785 (45.98)	1963 (32.41)	
		No anxiety/worry at all	A little bit anxiety/worry	A lot of anxiety/worry	
13. Not having anxieties/worries	6088	1670 (27.43)	2793 (45.88)	1625 (26.69)	

Regarding the positive relationships with friends and school teachers, 70% of the children indicated “very much” for “playing with friends a lot”, and 80% of the children indicated “very much” for “getting along well with friends”. More than 85% of the children perceived that other children liked them (“very much” and “a little bit”), school teachers approved of their efforts (“very much” and “a little bit”), and they were happy when talking to school teachers (“very much” and “a little bit”).

Regarding the positive perceptions of current circumstances, more than 80% of the children perceived that they were able to prepare for classes independently (“very well” and “fairly”), were proud of themselves (“very well” and “a little bit”), and were helpful to others (“very well” and “a little bit”). More than 95% of the children believed their efforts would be rewarded (“very well” and “a little bit”).

For the items on health status, 95% of the children perceived their subjective health status positively (“good” and “fair”). Only 20% of the children “did not get tired at all” while 80% of the children perceived becoming tired (“sometimes” and “frequently”). Only 30% of the children “had no anxiety/worry at all” while 70% of the children perceived having anxieties/worries (“a little bit” and “a lot”).

Table 3 shows the response characteristics regarding the persons the children talked to when sharing experiences and thoughts with. More than 90% of the children indicated doing so with their mother (“frequently” and “sometimes”). More than 70% of the children indicated doing so with their father (“frequently” and “sometimes”). More than 60% of the children indicated doing so with siblings, other family members/relatives, and school teachers (“frequently” and “sometimes”). For school counselors, nearly 50% of the children indicated “rarely” and more than 40% of the children indicated “no one such person”. For instructors of extracurricular activities, more than 40% of the children indicated “rarely”. More than 80% of the children indicated doing so with friends (“frequently” and “sometimes”).

**Table 3:** Response characteristics regarding the persons the children talked to when sharing experiences and thoughts with according to response alternatives

Items	Valid response N	Response alternatives N (%)			
		Frequently	Sometimes	Rarely	No one such person
a. Mother	6099	4274 (70.08)	1484 (24.33)	284 (4.66)	57 (0.93)
b. Father	6036	2144 (35.52)	2399 (39.74)	1104 (18.29)	389 (6.44)
c. Siblings	6020	2104 (34.95)	1521 (25.27)	1603 (26.63)	792 (13.16)
d. Other family members/relatives	6068	1370 (22.58)	2362 (38.93)	2069 (34.10)	267 (4.40)
e. School teachers	6074	1139 (18.75)	2611 (42.99)	2257 (37.16)	67 (1.10)
f. A school counselor	6006	72 (1.20)	362 (6.03)	2937 (48.90)	2635 (43.87)
g. Instructors of extracurricular activities	6079	1104 (18.16)	1404 (23.10)	2681 (44.10)	890 (14.64)
h. Friends	6100	3622 (59.38)	1675 (27.46)	746 (12.23)	57 (0.93)

Regarding the comparisons of response characteristics between boys and girls, all question items indicated an ignorable level or no association in the comparison, indicating no difference in responses between boys and girls.

Table 4 shows the values for correlation coefficients between variables. No variable showed significant and positive correlation with how children felt about attending school (“I am looking forward to going to school”) at an excellent or moderate level. The following variables showed significant and positive correlations with how children felt about attending school at a fair level: “getting along well with friends” (*r* = 0.303), “other kids like me” (*r* = 0.306), “school teachers approve of my efforts” (*r* = 0.339), “being happy when talking to school teachers” (*r* = 0.428), “being able to prepare for classes by own” (*r* = 0.254), “being proud of oneself” (*r* = 0.320), “being helpful to others” (*r* = 0.314), “believing one’s efforts will be rewarded” (*r* = 0.306), and “subjective health status” (*r* = 0.273). One significant and positive correlation at a moderate level was observed between “being proud of oneself” and “being helpful to others” (*r* = 0.526).

**Table 4:** Spearman’s rank correlation coefficient between variables

	1	2	3	4	5	6	7	8	9	10	11	12	13	a	b	c	d	e	f	g	h
1	1	0.247*	0.303*	0.306*	0.339*	0.428*	0.254*	0.320*	0.314*	0.306*	0.273*	0.175*	0.139*	0.148*	0.146*	0.086*	0.134*	0.210*	0.004	0.118*	0.179*
2		1	0.423*	0.316*	0.205*	0.194*	0.066*	0.214*	0.184*	0.198*	0.183*	0.132*	0.102*	0.102*	0.083*	0.072*	0.110*	0.122*	− 0.015	0.103*	0.231*
3			1	0.409*	0.257*	0.218*	0.145*	0.244*	0.257*	0.211*	0.213*	0.110*	0.120*	0.148*	0.121*	0.107*	0.103*	0.091*	0.003	0.107*	0.252*
4				1	0.458*	0.291*	0.206*	0.357*	0.407*	0.260*	0.228*	0.147*	0.118*	0.140*	0.100*	0.092*	0.104*	0.109*	− 0.008	0.095*	0.199*
5					1	0.458*	0.258*	0.397*	0.438*	0.297*	0.222*	0.130*	0.064*	0.169*	0.143*	0.088*	0.125*	0.243*	− 0.001	0.118*	0.137*
6						1	0.158*	0.280*	0.309*	0.286*	0.180*	0.076*	0.022	0.157*	0.147*	0.078*	0.133*	0.348*	− 0.002	0.102*	0.152*
7							1	0.201*	0.249*	0.176*	0.179*	0.106*	0.091*	0.112*	0.057*	0.060*	0.039*	0.045*	0.023	0.070*	0.074*
8								1	0.526*	0.414*	0.255*	0.107*	0.112*	0.152*	0.172*	0.117*	0.143*	0.182*	0.033**	0.181*	0.170*
9									1	0.363*	0.247*	0.109*	0.058*	0.189*	0.166*	0.134*	0.145*	0.180*	0.024*	0.164*	0.151*
10										1	0.224*	0.074*	0.043*	0.187*	0.164*	0.084*	0.122*	0.160*	0.010	0.130*	0.140*
11											1	0.195*	0.161*	0.159*	0.123*	0.101*	0.093*	0.120*	0.004	0.114*	0.129*
12												1	0.265*	0.029	0.054*	0.017	0.040*	0.022	− 0.017	0.013	0.013
13													1	0.016	0.055*	0.038*	0.037*	0.015	− 0.014	0.040*	0.031
a														1	0.399*	0.301*	0.322*	0.303*	− 0.054*	0.247*	0.312*
b															1	0.341*	0.361*	0.331*	− 0.039*	0.287*	0.295*
c																1	0.315*	0.326*	− 0.013	0.281*	0.343*
d																	1	0.257*	− 0.054*	0.311*	0.353*
e																		1	− 0.047*	0.353*	0.391*
f																			1	0.170*	− 0.042*
g																				1	0.340*
h																					1

^*^p < 0.001

1: Looking forward to going to my school; 2: Playing with friends a lot; 3: Getting along well with friends; 4: Other kids like me; 5: School teachers approve my efforts; 6: Being happy when talking to school teachers; 7: Preparing for classes by own; 8: Being proud of oneself; 9: Being helpful to others; 10: Believing one’s efforts will be rewarded; 11: Subjective health status; 12: Not getting tired easily; 13: Not having anxieties/worries; a: mother; b: father; c: siblings; d: other family members/relatives; e: school teachers; f: school counselors; g: instructors of extracurricular activities; h: friends for Persons I talk to when sharing experiences and thoughts

### The initial model examination

**Fig. 2:**
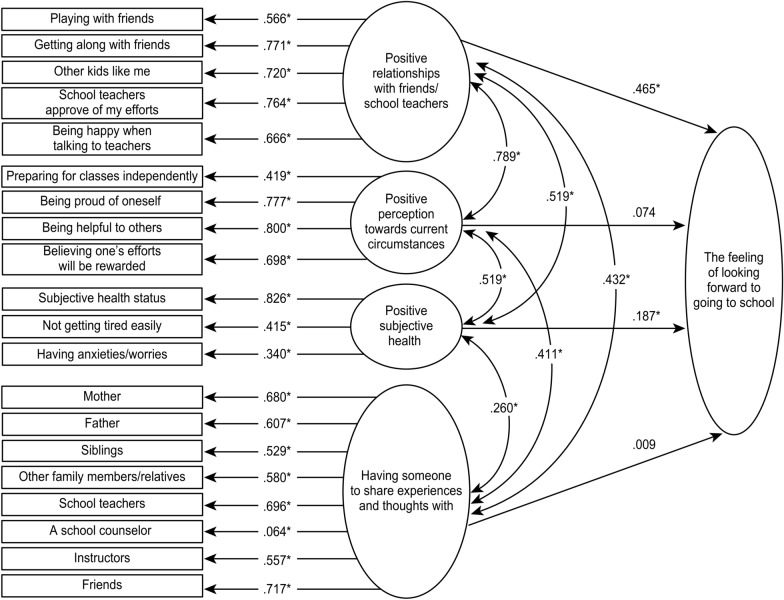
The first examination using a SEM. Numbers are path coefficients, *p < 0.001

Figure 2 shows the first examination using a SEM. The chi-square test of model fit was 4066.546 (degrees of freedom was 180, *p* < 0.001). Model fit indices were 0.056 for RMSEA (90% CI: 0.055, 0.058), 0.931 for CFI, and 0.919 for TLI. Positive relationships with friends and school teachers (0.465 for the path coefficient value, *p* < 0.001) and positive subjective health (0.187 for the path coefficient value, *p* < 0.001) directly impacted how children felt about attending school. The latent variables of positive perception of current circumstances (0.074 for the path coefficient value, *p* = 0.014) and having someone to share experiences and thoughts with (0.009 for the path coefficient value, p = 0.534) were not found to be related significantly to how children felt about attending school.

Among the latent variables, the following relations were observed. Positive relationships with friends and school teachers was positively and significantly associated with a positive perception of current circumstances (0.789 for the path coefficient value), positive subjective health (0.519) and having someone to share experiences and thoughts (0.432) (all *p* < 0.001). A positive perception of current circumstances was positively and significantly associated with positive subjective health (0.519 for the path coefficient value) and having someone to share experiences and thoughts (0.411) (both *p* < 0.001). Positive subjective health was positively and significantly associated with having someone to share experiences and thoughts (0.260 for the path coefficient value, *p* < 0.001).

For the latent variable of positive relationships with friends and school teachers, the path coefficient values for the variables were between 0.764 and 0.566 (all *p* < 0.001). For the latent variable of a positive perception of current circumstances, the path coefficient values for the variables were between 0.800 and 0.419 (all *p* < 0.001). For the latent variable of positive subjective health, the path coefficient values for the variables were between 0.826 and 0.340 (all p < 0.001). For the latent variable of having someone to share experiences and thoughts, the path coefficient values for variables were between 0.717 and 0.529 (all *p* < 0.001). A school counselor was an exception, showing a low path coefficient value (0.064, *p* < 0.001). We excluded the school counselor in the model modification.

### Model modification

**Fig. 3:**
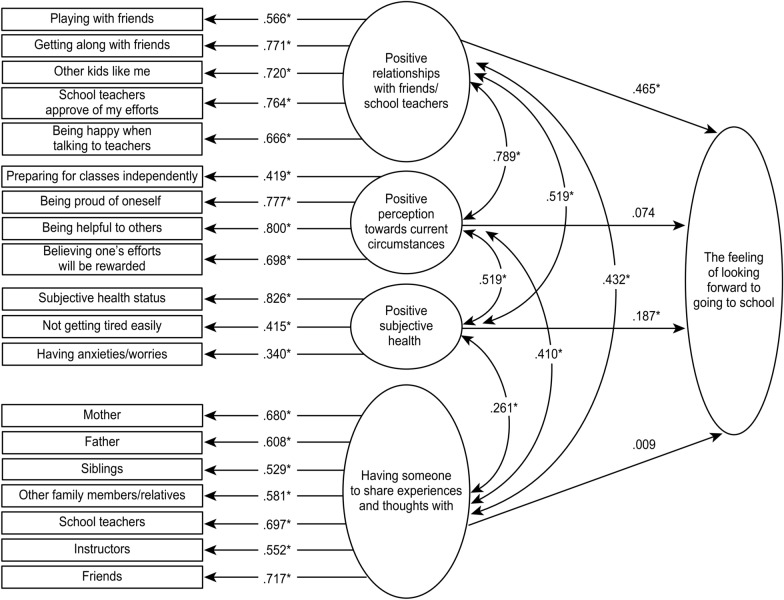
Modified model using SEM. Numbers are path coefficients, *p < 0.001

Figure 3 shows the modified model using SEM. Chi-square test of model fit was 3913.983 (degrees of freedom was 181, *p* < 0.001). Model fit indices were 0.0055 for RMSEA (90% CI: 0.053, 0.056), 0.933 for CFI, and 0.923 for TLI. Compared with the first examination, the values for the model fit indices slightly improved, but the values obtained for the path coefficient were almost the same as the values in the first examination. The modified model was selected to describe the structural relation of how children felt about going to school.

## Discussion

This study identified the structural relations among positive perceptions of attending school for elementary school students in the 5th year in state-run schools in Saitama, Japan. The latent variable of positive relationships with friends and school teachers was positively and directly associated with children’s feelings towards attending school. Previous studies emphasized the importance of positive relationships with friends and teachers for overcoming school nonattendance [11–24]. This study found that these relationships were important for positive perceptions of attending school for elementary school students in the 5th year. Other latent variables did not influence feelings towards attending school strongly or did not impact it at all; however, those latent variables were positively and significantly associated with the latent variable of positive relationships with friends and school teachers. Some individual items (e.g., “being able to prepare for classes by own”, “being proud of oneself”, “being helpful to others”, “believing one’s efforts will be rewarded”, and “subjective health status”) indicated significant correlations with feelings towards attending school. Future studies may provide possible solutions for refining the questionnaire.

It may be important to consider positive subjective health with greater regard to psychological health. Peer acceptance in school is associated with subjective health [41]. Support from school teachers and classmates has also been found to be significantly and positively related to “school satisfaction” [42] and emotional stability [43] among elementary school students. Feelings of being supported by adults have been identified as important for children with social, emotional and mental health-related difficulties to flourish in the school environment [44]. Teachers have the potential to improve and intervene in students’ mental health [45]. A range of interventions have been tested for mental health promotion in schools with varying degrees of success [46]. Evidence-based strategies to improve positive emotions and well-being may be useful in supporting children who have issues with attendance [47–50]. There was an item of “having anxieties and/or worries” in this study with 73% of children either stating “a little bit” or “a lot” anxieties and/or worries. Internalized mental health issues with high frequencies in children should be examined in future studies.

This study highlighted that more than 60% of the children in this study indicated that school teachers were the people to whom the children talked when sharing experiences and thoughts, a frequency that was equivalent to siblings and other family members. It implies that school teachers were perceived as accessible and available adults. For reducing the burden of school teachers, school counselors have recently been introduced in Japan [17]; however, the presence or absence of school counselors was not found to be important in this study. The employment system of counselors, i.e., being employed directly by school district offices but not in schools may account for this result. For elementary school students, specialist counsellors were not as accessible as other staff, such as teachers.

### Strengths and limitations of the study

A large random sample, obtained by collaboration with the educational board in Saitama, Japan, enabled us to examine our hypothesized model robustly. An excellent response rate was supported by the school teachers who distributed and collected the original questionnaire.

However, this study has some limitations. The first issue is the reliability of the original questionnaire although it was developed based on previous studies [1–16, 18–24] and national guidelines [17], as well as previous piloting. The current study was the first attempt to examine the questionnaire with a large sample and explore the hypothesized model. There may be other variables that were not included that account for some of the variance in the outcomes. Additionally, the generation of items was based on a rigorous development process; however, children were not included in development. Future studies may replace the current variables or add variables to improve the model fit indices. Methods including students may be used to improve the questionnaire. The second issue was missing information regarding whether students with a non-school attendance history were included in this study. Thus, we were unable to compare responses between students with poor versus good attendance. In future studies, asking whether students have a non-school attendance history and whether they have obtained support to overcome it, may be needed. The third issue was a homogeneity of the students in terms of school setting and age rage. Thus, the model has to be examined in different settings and a wider age range.

## Conclusions

This study examined children’s perspectives on school attendance and related variables through a survey of 6841 students in the 5th year of state-run elementary schools. This study found that positive relationships with friends and school teachers were significantly and positively associated with children looking forward to attending school. Positive health status was also significant, but the relationship was not as strong. This study explored determinants of school attendance by using a novel questionnaire in Japan and reported the findings by presenting an original model.

## Supplementary Information

**Additional file 1: Table S1.** Demographic characteristic of school districts.

## Data Availability

The data are stored at the Saitama Educational Board office and restricted for research use only before April 2022. The data are not publicly available. Please contact the corresponding author to discuss data access.

## Abbreviations

CFI: Comparative fit index
ICC: Intraclass correlation coefficient
RMSEA: Root mean square error of approximation
SEM: A structural equation model
TLI: Tucker Lewis index
WLSMV: Weighted least squares method
