# Supplementary material for: Determinants of school attendance in elementary school students in Japan: a structural equation model
Source: Child Adolesc Psychiatry Ment Health. 2021 Jul 27;15:38. doi: 10.1186/s13034-021-00391-5 (PMC8317317; doi:10.1186/s13034-021-00391-5)
Supplement: Supplementary file 1 — Additional file 1: Table S1. Demographic characteristic of school districts. [file 13034_2021_391_MOESM1_ESM.docx]

| **Additional file**  **Table S1** Demographic characteristic of school districts | | | | | | | | | |
| --- | --- | --- | --- | --- | --- | --- | --- | --- | --- |
| Demographic characteristics | Total | School district | | | | | | | |
|  |  | A | B | C | D | E | F | G | H |
| Public elementary schools | 111 | 29 | 12 | 15 | 17 | 4 | 6 | 16 | 12 |
| Public elementary school students | 48190 | 9556 | 3861 | 6784 | 8995 | 1461 | 2168 | 7301 | 8064 |
| 5th year students belong to the schools | 8045 | 1539 | 641 | 1139 | 1512 | 247 | 351 | 1285 | 1331 |
| Collection for this study | 6860 | 1468 | 530 | 928 | 1193 | 197 | 311 | 1020 | 1213 |
| Analyzed data in this study | 6841 | 1465 | 530 | 925 | 1189 | 197 | 307 | 1015 | 1213 |
| School counselors | 14 | 3 | 1 | 3 | 3 | 0 | 0 | 1 | 3 |
| Population |  | 196,800 | 77,800 | 150,800 | 164,000 | 33,900 | 44,700 | 148,400 | 140,600 |
|  | | | | | | | | | |
